# Chikamatsu, Mori, and the uncanny valley

**DOI:** 10.1177/20416695251317469

**Published:** 2025-02-06

**Authors:** Karl F. MacDorman

**Affiliations:** Indiana University, Indianapolis, IN, USA

**Keywords:** anthropomorphism, *bunraku*, humanoid robotics, *jōruri*, realism

## Abstract

In Japan, robotics projects like Geminoid, modeled after Hiroshi Ishiguro, exhibit a fascination with creating human doubles. Yet, warnings against this also thread through Japanese thought, from the Edo-period playwright Chikamatsu Monzaemon (1653–1724) to the robotics professor Mori Masahiro (1927–2025). Though centuries apart, they describe the same *uncanny valley* phenomenon—eerie, cold, repellent feelings that arise when confronting the imperfectly human. In an interview with Hozumi Ikan, translated here, Chikamatsu presents a theory of realism exemplified through puppet theater and kabuki. He divides realism into four zones: the unreal, conceptual realism, surface realism, and the real. The unreal lacks authenticity, surface realism lacks soul, and the real lacks expressiveness. For Chikamatsu, it is conceptual realism that captivates an audience. A play's unfolding events evoke empathy and emotion through their meaning for the characters. Similarly, Mori divides realism into four zones: industrial, humanoid, and android robots, and real people. Industrial robots evoke little affinity, and androids risk appearing eerie. Though real people evoke the most affinity, androids cannot become indistinguishable from them. For Mori, only humanoid robots evoke affinity without risking uncanniness. By exploring anthropomorphism, both Chikamatsu and Mori illuminate principles for designing robots that do not unsettle but delight.

## How to cite this article

MacDorman, K. F. (2025). Chikamatsu, Mori, and the uncanny valley. *i-Perception*, 16(0), 1–18. https://doi.org./10.1177/20416695251317469

## Introduction

**Figure 1.**
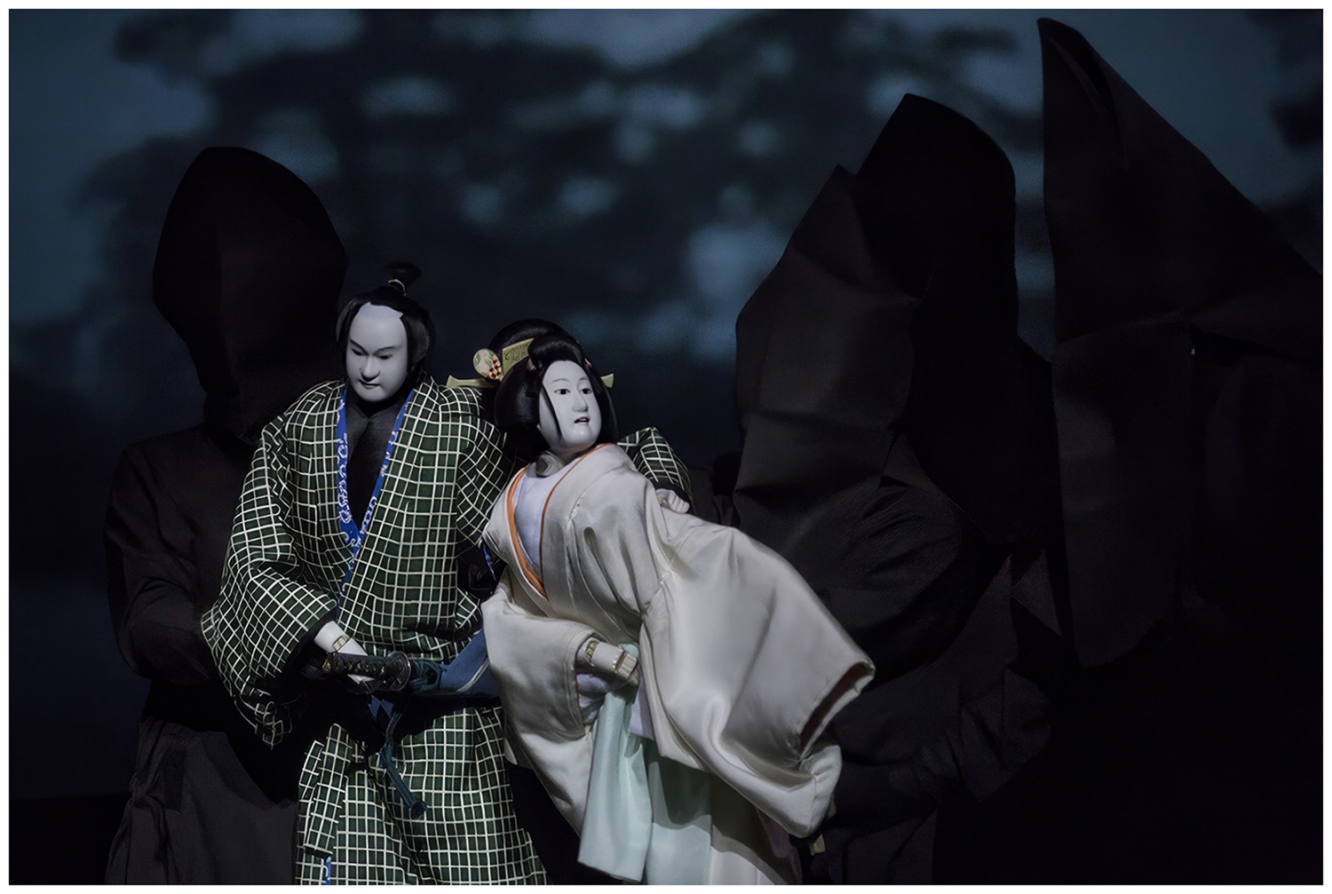
Hiranoya Tokubei and Temmaya Ohatsu in Chikamatsu Monzaemon’s *The Love Suicides at Sonezaki*, directed by Sugimoto Hiroshi, at the Théâtre de la Ville in Paris on October 9, 2013 (Photo credit: Odawara Art Foundation).

Chikamatsu Monzaemon^1^ (1653–1724) is Japan's most celebrated playwright. Dubbed the profession's guardian deity, he wrote at least 40 plays for kabuki and 114 for *ningyō jōruri*^2^ (hereafter, *jōruri*), a form of musical puppet theater he transformed and popularized (Kawatake, 1988; Figure 1). His only known work of theater criticism is reported in an interview with a close friend and *jōruri* devotee, the Confucian scholar Hozumi Ikan (1692–1769). This interview, published posthumously in the preface to Hozumi's (2016) *Naniwa Miyage*, presents Chikamatsu's theory of realism (Kan, 2019; Pastreich, 2011). His insights can inform contemporary discourse on character and interaction design in robotics and AI, especially as it relates to the *uncanny valley*.

The uncanny valley graphs how affinity for robots, dolls, and puppets plunges into eeriness when they begin to resemble humans too closely. The concept, first proposed in 1970 by the Japanese robotics professor Mori Masahiro (2012), has taken hold in the West this century. Beyond its influence on popular culture, a 2022 meta-analysis identified 488 papers on the uncanny valley, 354 of which included empirical studies; it revealed, based on 247 effects, that the phenomenon has a large effect size (Diel et al., 2022). Moreover, large-scale studies have reproduced the uncanny valley's characteristic shape.^3^

Theories explaining the uncanny valley span a range of disciplines, including perceptual, cognitive, social, and evolutionary psychology, neuroscience, anthropology, and philosophy of mind (MacDorman et al., 2009b; Wang et al., 2015). However, this abundance of theories complicates the task of weighing their plausibility and empirical support. Among these, Kätsyri et al. (2015) found evidence in the literature for theories predicting the source of the effect lies in perception, such as when an entity's features are mismatched in their level of animacy, human likeness, or realism (MacDorman & Chattopadhyay, 2016; MacDorman et al., 2009a; Mitchell et al., 2011; Moore, 2012; Seyama & Nagayama, 2007). Recent research also supports perceptual theories, indicating uncanny stimuli are processed quickly and effortlessly. For example, a 50-ms exposure to humanlike robots can elicit eeriness comparable to a 3-s exposure (Yam et al., 2024). Shin et al. (2019) found uncanny avatars are immediately assessed and rejected through an automatic process that suppresses further cognitive evaluation.^4^ Nevertheless, interpersonal inferences, such as attributions of mind, may intensify the uncanny valley effect (MacDorman, 2024).

Why does an entity with mismatched features—some human, some mechanical—appear uncanny? Mismatched features cause conflicting perceptions about what the entity is. If it looks human, opportunities for perceptions to conflict proliferate for several reasons. First, children develop from infancy expertise in recognizing human faces, hands, and bodies and their expressions, gestures, and poses. This process of perceptual narrowing sensitizes specialized brain areas to human appearance, rendering imperfections more salient (Chattopadhyay & MacDorman, 2016). Second, children acquire the ability to process human forms configurally. For example, recognizing the face's first-order features, like two eyes above the nose and mouth, scaffolds learning to recognize second-order features, like how widely set the eyes are. These second-order features become additional dimensions along which norms may be violated, increasing opportunities for perceptions to conflict (Diel & MacDorman, 2021). Norm violation as an explanation of the uncanny valley is consistent with neuroimaging results showing heightened N400 event-related potentials for incongruous feature combinations, such as nonbiological movement in a humanlike robot (MacDorman & Ishiguro, 2006; Urgen et al., 2018). Third, our humanity sets us in a particular relation to humanlike entities, causing us to feel greater sociality, empathy, attraction, and threat (MacDorman, 2019a; MacDorman et al., 2009b).

Much is known about what elicits the uncanny valley, but less about its effects (Diel et al., 2022). That is surprising, given what is at stake. Anecdotally, the uncanny valley has been faulted for box office flops and studio closures, as characters animated with human motion capture have appeared unsettling and emotionally alienating (Butler & Joschko, 2009; Freedman, 2012). The uncanny valley's economic impact, though unknown, could be staggering. In 2023, the global animation, visual effects, and video games market was valued at $259.3 billion and is projected to reach $563.6 billion by 2032 (Allied Market Research, 2024). Despite this, little is known about how the uncanny valley affects our appreciation of visual narratives (MacDorman, 2019). Thus, it is worth revisiting centuries-old insights from Japanese puppet theater. Chikamatsu's theory of realism explains how to employ conceptual realism to circumvent the uncanny valley while enhancing emotional engagement.

Conceptual realism creates an aura of realism by conveying the characters’ intentions, motivations, and emotions through action and dialogue instead of replicating their appearance. Narrative tension is created by throwing their desires and passions into conflict with their commitments and duties. Conceptual realism engenders empathy through the gravity of unfolding events for the characters, enabling the audience to experience the story as real. Symbolism and stylization set meaning above representation, depicting characters’ status, rank, and disposition to clarify their social relationships. Conceptual realism encourages departures from reality that enhance expressiveness and artistry.

Chikamatsu contrasts conceptual realism with surface realism, which replicates reality's outward appearance. This exactness results in a lack of emotional depth. The representation feels “vacant and eerie” because its proximity to human appearance exposes its absence of life or a “soul.” By focusing on superficial details, surface realism neglects the symbolic and emotional elements that engage the audience. Instead, they are left with the hollow, unsettling sensation, described by Mori (2012) in his 1970 essay on the uncanny valley. In contrast, conceptual realism avoids the uncanny valley by emphasizing emotional and narrative engagement.

In subsequent sections, I recount Chikamatsu's biography, translate his interview with Hozumi, reinterpret his theory of realism by relating it to the uncanny valley, and apply it to AI and robotics research. As a translator of Mori's essay and a researcher publishing extensively on the uncanny valley, I explore new connections between Chikamatsu's theory, human psychology, and contemporary robotics. My aim is to show the universality of Chikamatsu's insights, implicit in Mori's uncanny valley concept, in developing social robots. Today's designers share Chikamatsu's ambition to bring soulless creations to life, which is why his advice remains relevant.

## The Playwright's Life and Contribution

Chikamatsu was born Sugimori Nobumori, the second son of a samurai who served as a medical doctor under daimyo (lord) Matsudaira Tadamasa (Miyoshi, 2016). After losing his office, Chikamatsu's father moved to Kyoto. Chikamatsu briefly served as a page to the nobleman Ichijō Ekan, then entered *Gonshō-ji* monastery in Ōmi province, where he studied classical Japanese literature, music, court ceremony, etiquette, and Buddhism (Iwao et al., 1975).

**Figure 2.**
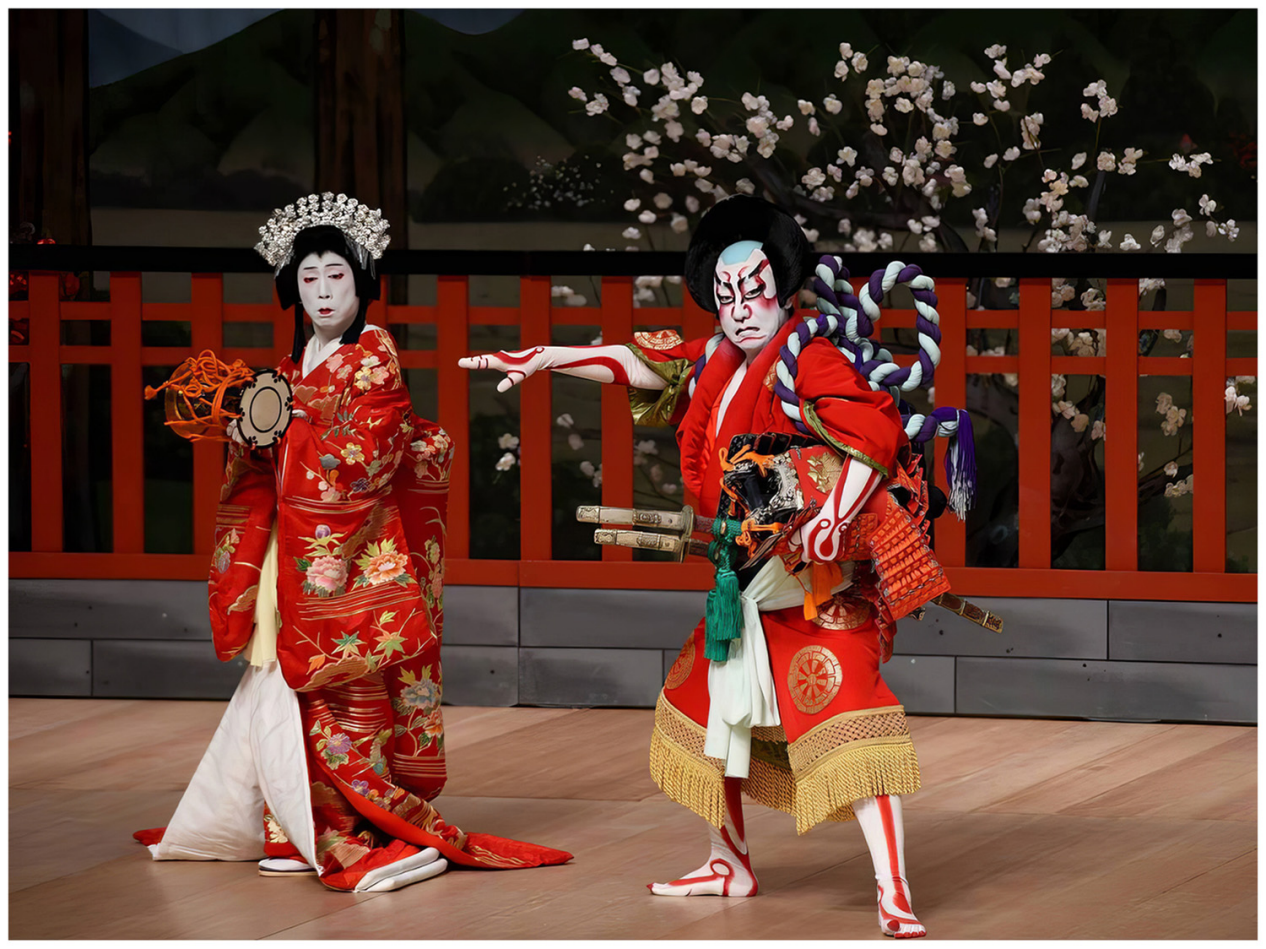
Kataoka Takatarō (left) and Shichidaime Nakamura Shikan (right) play Shizuka Gozen and Satō Tadanobu from *Yoshitsune and the Thousand Cherry Trees* at the Beijing Tianqiao Performing Arts Center on March 17, 2019. The script was originally written for *jōruri* by Takeda Izumo II, Miyoshi Shōraku, and Namiki Sōsuke (Photo credit: Top Photo/Alamy).

Chikamatsu started writing *jōruri* plays at 25 or 26 and continued for the rest of his life. In 1684, he began a 30-year collaboration with the narrator Takemoto Gidayū (1651–1714). Takemoto opened his theater in Osaka with *The Soga Heir*, which Chikamatsu had written for Uji Kaganojō's theater in Kyoto the year before. Beginning with Takemoto's production of *Kagekiyo Victorious* in 1685, set after the Genpei War (1180–1185), Chikamatsu innovated a new style of *jōruri* with more realistic, expressive, and riveting plots (Yoshikawa, 1965). Beyond *jōruri*, Chikamatsu wrote plays for kabuki, a form of musical theater with live actors known for stunning costumes, dramatic makeup, fierce poses (*mie*), and stylized dance (Figure 2). In 1706, when the kabuki actor Sakata Tōjūrō (1647–1709) retired, Chikamatsu moved to Osaka to dedicate himself to writing *jōruri* for Gidayū's theater (Kawatake, 1988).

The bulk of Chikamatsu's *jōruri* repertoire consists of 90 *jidaimono* (plays depicting historical events and figures). These period pieces include romances and epic battles involving nobles and samurai. Masterpieces of this genre include *The Snow Woman* (1705), *The Battles of Coxinga* (1715), and *The Soga Brothers at Kaikei Mountain* (1718). The remainder consists of 24 *sewamono* (plays depicting the contemporary life of ordinary people). These domestic dramas have received the most enduring acclaim (Chikamatsu, 1961). Set in the present, focused on family conflicts, sometimes featuring an antihero, and exposing life on society's fringes, they marked a transgressive departure from earlier *jōruri* (Gerstle, 1996). A character inspired by an orphan, miscreant, or murderer could leap from the headlines onto the stage. Chikamatsu's domestic dramas include *The Love Suicides at Sonezaki* (1703), *The Love Suicides at Amijima* (1720), and *The Woman-Killer and the Hell of Oil* (1721). In *Woman-Killer*, the oil merchant's son, Yohei, falls heavily into debt and turns to blackmail and murder. Chikamatsu's portrayal of Yohei as an antihero rather than a villain resonates with contemporary audiences.

Chikamatsu elevated puppet theater to the apex of dramatic art. His plays, the first to incorporate townspeople as a literary genre, along with similar stories by poet and novelist Ihara Saikaku (1642–1693), continue to shape Japanese literature. His domestic dramas, known for their acute insights into family and romantic relationships, explore the tension between desire and duty (Iwao et al., 1975). That this tension nearly always resolves in tragedy indicts the rigid feudal order, unable to fulfill individual needs in an urbanizing society.

My translation of Hozumi's interview with Chikamatsu follows.

## *Preface to* Naniwa Miyage *by Hozumi Ikan*

Some years ago, when I visited Chikamatsu, this is what he told me:

1. *Jōruri* differs from other forms of storytelling. As the puppets’ role is paramount, every verse must spring to life through movement. Moreover, *jōruri* vies for patrons with nearby kabuki theaters and the artistry of their live actors. To move the audience, the playwright must evoke various emotions using soulless wooden dolls. Without extraordinary effort, it would be hard to write a play of great renown.
  Luckily, while reading *The Tale of Genji*^5^ in my youth, I stumbled upon this passage: “At the time of a seasonal court feast, snow had fallen heavily, blanketing a *tachibana* tree.^6^ A guard was dispatched, but no sooner had he brushed off the *tachibana* than the pine tree beside it, still bending under the weight of snow, recoiled its branches with reproach.” With these strokes of a writing brush, the author breathed life into a soulless plant. That is because the pine tree, envying the care the tachibana had received, shook the snow from its own branches. Does this not create the impression of a being coming to life?
  This passage made me realize how to imbue my *jōruri* with soul. Descriptions of scenery,^7^ not to mention narration and dialogue, must be steeped in emotion, lest the work fail to stir deeply. This is akin to what poets call *evocative imagery.* For instance, when extolling in poetry the magnificent landscapes of Matsushima or Miyajima,^8^ unless one writes with exaltation, it would be like gazing inattentively at a beautiful woman's portrait. That is why each phrase must be rooted in emotion.
2. Whatever the verse, using too many particles like *te* [and], *ni* [to], and *ha* [as for] somehow degrades it. Yet untalented writers, confusing *jōruri* with poetic forms like *waka* and *haikai,*^9^ try to fit their verses into a 5–7 syllable pattern, resulting in superfluous particles. For example, if they should write *Toshi mo yukanu musume wo* [a girl not yet come of age], they instead write *Toshiha mo yukanu, musume wo ba* [as for a girl not yet come of age, indeed]*.* This fixation with counting syllables makes the dialogue sound unrefined. Though short and long lines are arranged in poetry, *jōruri* consists of song with string accompaniment, so the melody determines each line's length. Should a playwright adhere strictly to poetic meter, the verses could prove awkward to chant. As I have not attempted this, my scripts contain few particles.^10^
3. *Jōruri* used to be like today's *saimon* [street storytelling and narrative ballads]*.* It lacked substance and flair. After leaving Uji Kaganojō's troupe to write plays for Takemoto Gidayū, I chose my words with great care to lift *jōruri* to a high art. For example, the nobility, samurai, and lower tiers have distinct ranks and social positions, and each must be depicted accordingly in speech and manner. Even within the samurai, the daimyo, *karō* [chief retainer], and lower ranks, whose stipends vary, must be distinguished. This ensures that the audience empathizes with each character.
4. *Jōruri* depicts life as it happens. But in the interest of art, it also exposes hidden aspects. It is standard practice in kabuki for an *oyama* [male actor who portrays female roles] to deliver lines that a real woman would not utter. By talking openly, the character reveals her true feelings and circumstances. If a playwright were instead to model the character on a real woman, thereby concealing her motives and intentions, the performance—in all its accuracy—would fail to delight. Lines unbefitting a woman invite criticism from those who overlook the need for this device, but such departures from realism should be viewed as art. Likewise, when a villain acts too cowardly or a fool too buffoonishly, this should be viewed as art rather than a presentation of reality, and the audience should accept it as such.
5. Writers, declaring melancholy to be essential to *jōruri,* pepper their plays with expressions like *How sorrowful!*^11^ Otherwise, they have the lines chanted in a tear-drenched voice in the *bunya-bushi* style.^12^ These methods are absent from my work. In my plays, sorrow arises solely from each character's adherence to duty. As the plot thickens, the burdens of duty intensify. The melody and dialogue are so tightly constructed that the audience is deeply moved. To lament “How sorrowful!” distracts from its causes, diminishing the final impression. Sorrow must arise, not on command, but spontaneously.
  Furthermore, to admire a landscape like Matsushima in a single breath, “Ah! What a splendid view!” gives the impression of there being nothing left to say, thus diminishing it. To praise the scenery, take instead an indirect approach: Point out several of its features to let its appeal be grasped naturally. This advice holds for anything of this kind.
6. Hozumi remarked: I have heard it said that society these days [early 18th-century Japan] will not accept things unless they seem well reasoned, realistic, and convincing. Many aspects of old stories no longer persuade. They deem kabuki actors skillful only if their performances resemble real life. An actor portraying a *karō* must resemble a real *karō*, one portraying a daimyo must resemble a real daimyo, and so on. Audiences will not tolerate, as they had in the past, the kinds of artifice that would only fool a child.
  Chikamatsu answered: This argument may sound plausible but shows no grasp of the essence of art. Art abides in the narrow gap between the flesh and the skin, the real and the unreal. With today's preference for realism, an actor portraying a *karō* is expected to mimic the speech and gestures of a real *karō*. But does that mean a daimyo's *karō* should smear his face with powder and paint like the actor? Or, granted that his *karō* wears no makeup, would it then prove as entertaining to watch the actor perform on stage unpainted with a bald head and unkempt beard? This is what I mean by the narrow gap between the flesh and the skin: unreal yet not unreal, real yet not real. Somewhere between the two, amusement unfolds.
  This observation relates to the story of a court lady who became enamored with a man. Their passion was mutual, but the man could not call on her, as she was secluded deep within the imperial palace. She could only catch chance glimpses of him at court by peering through slits in the bamboo blinds. She was so madly in love with him that she ordered a wooden statue to be carved in his likeness. This was no ordinary doll. Its face and body differed not a hair's breadth from the man's. The coloration of his complexion, the pores of his skin, his earholes, nostrils, and mouth, and even the number of teeth inside, down to the tiniest detail, were copied exactly. Indeed, since the man had posed beside the figure while it was being made, it differed from him only in its absence of a soul. Yet when the court lady drew near to see it, though the doll was an exact replica, her enthusiasm instantly cooled. She somehow found it vacant and eerie. Her passion for the man cooled as well. Just having the doll around proved so disturbing that she soon discarded it.
  Reflecting on this, if we reproduce exactly a being of flesh and blood, even the ravishing Yang Guifei, at some point, our fondness runs out.^13^ Thus, whether painting a figure or carving it in wood, the artist must use broad strokes in places, even while aiming to stay true to its form, as it is a fabrication. Human likeness of this type is, after all, what inspires people's love. Likewise, in composing a plot, the playwright must use broad strokes in places while still following the original story, for this skill ultimately delights the audience. One should also keep this in mind when composing theatrical dialogue and the like.

## Chikamatsu's Divisions of Realism

Chikamatsu is often admired in film circles for proposing a theory of realism (Kan, 2019). On this view, the scriptwriter should not aim for surface realism—the reproduction of reality with utmost accuracy. Surface realism implies a set largely free of fancy costumes, wigs, and makeup. It would overthrow *jōruri* and kabuki, highly stylized artforms with traditionally established ways of appearing and acting, passed down through generations to convey emotions, character traits, and narrative elements.

Instead, Chikamatsu urges the scriptwriter to aim for conceptual realism, which upholds the abstract order behind reality to give meaning to events on the stage. Characters should be separated and ordered to reflect their role and status, with differences depicted through demeanor, gesture, and attire. In his domestic dramas, where societal expectations of duty and honor clash with human passions, conceptual realism emphasizes emotions, motivations, and societal roles over external likeness to real people. Paradoxically, stylization and symbolism are used to convey authenticity and emotional depth.

Conceptual realism, according to Chikamatsu, can enable the audience to identify with characters and sympathize with them. A play with conceptual realism—combined with expressive imagery and a compelling plot—will evoke the right feeling at the right time (Mangan, 2015). Joy and sorrow will arise from what the events unfolding on stage mean for each character, as the character's hopes and desires are aroused and thwarted. This progression of plot-driven emotions holds the audience in suspense and transforms the cast's actions into art. The performance need not be highly realistic, but the audience must experience it as meaningful because meaning makes the performance *feel* real.

Chikamatsu's theory states that art dwells in a narrow gap between the unreal and the real, the inauthentic and the uncanny. However, this metaphor should not be taken too literally (Lee, 2000). It reflects one powerful style of art. With the benefit of hindsight, we see that other styles do not abide in a narrow gap but in a wide expanse, from the abstract, geometric patterns of Oskar Fischinger's *An Optical Poem* (1937, echoed in Disney's *Fantasia*, 1940) to the gritty realism of Vittorio De Sica's *Ladri di Biciclette* (1948), shot on location with ordinary townsfolk. At the low end of realism, the movements of two triangles and a circle tell a story that exhibits their different personalities and lets viewers feel empathy for them (Heider & Simmel, 1944); at the high end of realism, security camera footage amuses viewers on social media. None of these examples feels fake; none feels eerie. Thus, we can experience art and meaning at any level of surface realism—even when venturing into the uncanny valley. A striking example is the play *Sayonara*, which features Hiroshi Ishiguro's Geminoid F and is directed by Hirata Oriza in a style of realism that meticulously captures daily life “as it is” (Poulton, 2023).

Nevertheless, Chikamatsu's theory of realism rings true for *jōruri*, kabuki, and social robotics for two reasons. First, Chikamatsu's aim was not just to create art but *popular* art. To this end, he employed conceptual realism to stir the passions. Unlike *nō*, *jōruri* and kabuki were the popular theater arts of his day. The stage was ablaze with action and drama, which, for kabuki, sometimes sparked a riot (Mezur, 2005; Ortolani, 1995). While Chikamatsu's new *jōruri* exhibited artistic merit, evidenced by its critical acclaim, he also multiplied the art form's popularity. Consider, for example, *The Battles of Coxinga*, which depicted Prince Coxinga's resistance against the Manchu conquest. At a time when a play might typically run for a few weeks, it ran for an unprecedented 17 months. Shakespeare, whose plays were also hugely popular in his day, similarly used conceptual realism in characterization: “Shakespeare does not paint individuals but individualizes classes. [He distinguishes in each character] the signs of a class of nature, midway between his general nature and his individual peculiarities” (Whipple, 1888, p. 49). Both Chikamatsu and Shakespeare were popular because they used conceptual realism to create archetypical and relatable characters and holistic and moving stories.

Second, Chikamatsu's theory rings true for *jōruri* and kabuki because conventions of symbolism and stylization are integral to these art forms and limit their surface realism. For today's youth in Japan, these conventions may obscure meaning, especially when combined with situations, social norms, and language made alien by the passage of time. In the United States, *kabuki theater* is a metaphor for elaborate but empty political posturing that leaves matters unresolved. Ironically, this is the opposite of how kabuki—or *jōruri*—would have been experienced during the Genroku period (1688–1704) and successive decades. Conflicts on stage were resolved, often tragically, by the action and dialogue, which the audience could follow because the situations, social norms, and language were still contemporary. Conventions clarified a play's meaning. By supporting conceptual realism, *symbolism* and *stylization* enhanced the play's popular appeal.

Symbolism and stylization have also been used to this effect in robot performances in Europe, Japan, and the United States (Sone, 2017; Sovhyra, 2021). For example, R2-D2 from *Star Wars*, employed distinctly nonhuman sounds, movements, and visual cues to convey personality and emotion.

**Figure 3.**
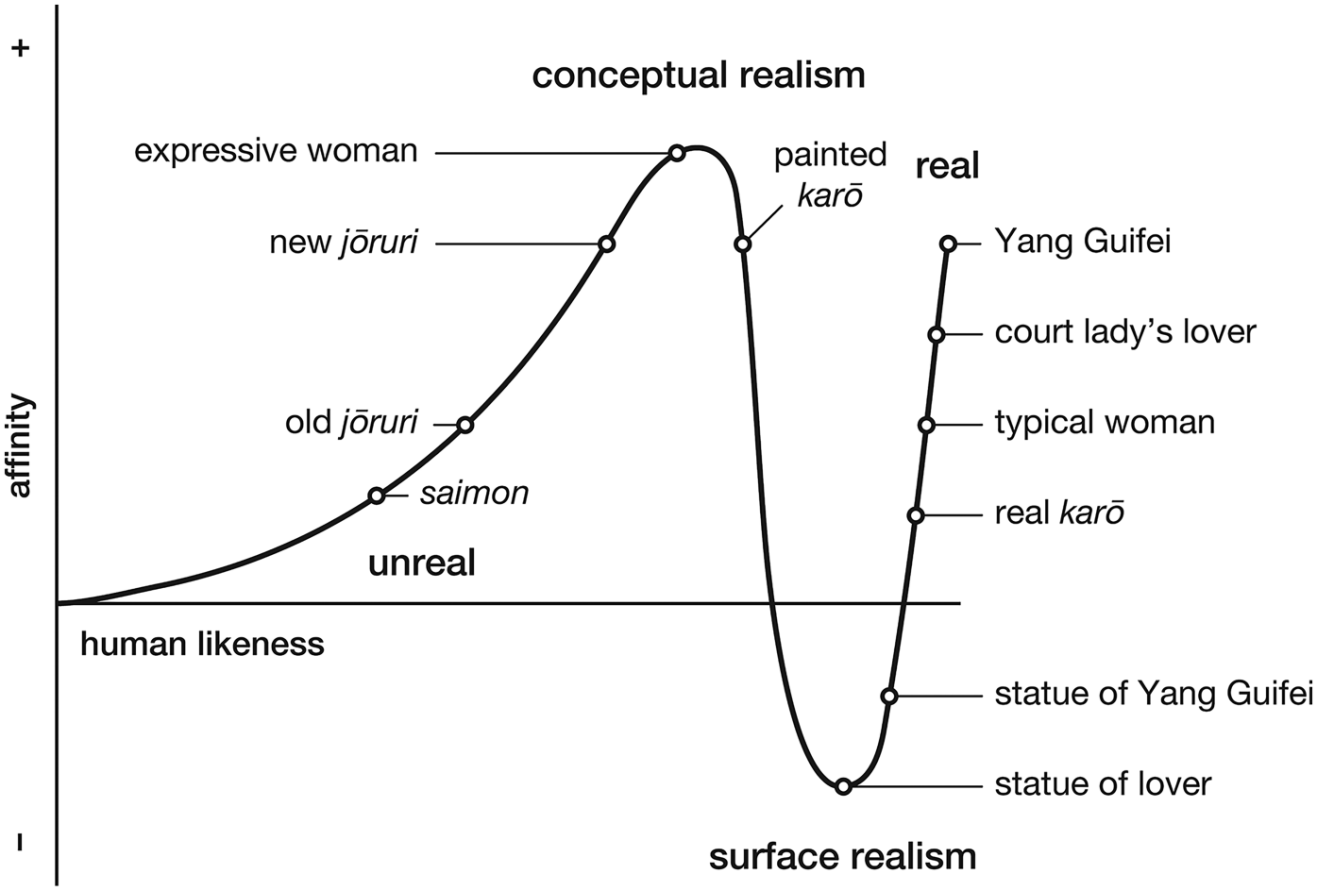
Chikamatsu's theater criticism illustrates how conceptual realism heightens affinity for characters through characterization and plot-driven emotions. Surface realism employs exact reproductions of reality, which are eerie. The audience will often prefer conceptual realism because its departures from reality can heighten a play's expressiveness and artistic merit.

The juxtaposition Chikamatsu makes between conceptual and surface realism, both of which he placed between the unreal and the real, may be interpreted as forming a graph (Figure 3). The unreal lacks authenticity and refinement, exemplified by the old *jōruri*, which echoes *saimon*'s street storytelling and narrative ballads. Surface realism lacks soul, exemplified by the “vacant and eerie” statue of the court lady's lover. The real lacks expressiveness, exemplified by the Edo period woman's stifled emotions and the real *karō*'s drab, disheveled appearance. As explained below, for Chikamatsu, conceptual realism weaves together authenticity, refinement, soul, and expressiveness to captivate the audience.

As indicated, the old *jōruri* was unrealistic and unconvincing. It lacked authenticity, relying on graceless theatrics. The narrator would tell the audience what to think and feel about a landscape. Then, from the story of the envious pine in *The Tale of Genji*, Chikamatsu learned how to enliven his puppets with emotion. Movement, dialogue, and evocative imagery would show events unfolding, letting the audience draw their own conclusions. The drama would hold the audience under its spell, free of the jarring proclamations of an overwrought narrator. In essence, the dramatic technique “show, don’t tell,” attributed to Anton Chekhov (1860–1904), had been proposed by Chikamatsu two centuries before it was popularized in the West (Lubbock, 1921). This technique was essential to his new *jōruri*.

Chikamatsu also contrasts real life with kabuki's conceptual realism: During the Edo period, a real woman was expected to keep her feelings buried, while a female character could express them openly, thus advancing the plot. Like a Shakespearean soliloquy, though unrealistic, this openness lets the audience understand the character's state of mind. Men were also given little latitude for free expression under the rigid feudal order of Edo period Japan. Neo-Confucianism, which permeated the educational system, valued hierarchy, conformity, propriety, and restraint over personal expressiveness. By contrast, kabuki enabled villains and fools to be portrayed with a level of exaggeration that would be sanctioned in real life. As is common in drama, the audience is expected to suspend disbelief.

Similarly, though a real *karō* presents his face without makeup, a kabuki actor could use makeup to portray the *karō's* disposition and state of mind. For example, the colors red and blue represent good and evil, respectively; lines on the face, hands, and forearms represent blood vessels throbbing with anger or passion; makeup shows whether the character is bold or reticent, thoughtful or reckless; and clothing indicates the character's social status. Though one might think the stylized kabuki versions of the woman and *karō* were false, Chikamatsu affirms that these exuberant departures from reality are essential to art. Thus, the roles of woman and *karō* are placed near the first peak in the graph.

Chikamatsu then sets up another contrast—this time between surface realism and real life: The court lady is unnerved by the statue of her lover, rendered with uncanny accuracy. Even a statue of Yang Guifei, whose beauty was legendary, would quell the viewer's affection if reproduced precisely. With these parables, Chikamatsu reaffirms that art sometimes demands departures from reality to enhance characters’ expressiveness and avoid eeriness.

## Conceptual Realism in Mori's Uncanny Valley

In today's Japan, *jōruri* and kabuki have been crowded out by other forms of entertainment (Poulton, 2023). Puppet theater is a dying art in the West as well.

Chikamatsu, however, is making broader observations about visual storytelling and the aesthetics of anthropomorphism. These observations apply to new forms of media like computer animation and virtual and augmented reality. Given Chikamatsu's goal—to create emotional engagement by breathing life into soulless puppets—his observations on *jōruri* apply especially well to social and humanoid robots.

The aesthetics of these robots were addressed in a 1970 essay by Mori Masahiro (2012), then a professor at the Tokyo Institute of Technology. Chikamatsu's theory of realism foreshadows Mori's uncanny valley concept, aligning with it in thought-provoking ways (Poulton, 2014). However, Mori's inspiration came not from Chikamatsu's theory but from the unsettling realism of myoelectric hands (Kageki, 2012). Their creepiness, along with his lifelong aversion to wax figures, prompted him to reflect on robots more generally, leading to his seminal essay.

**Figure 4.**
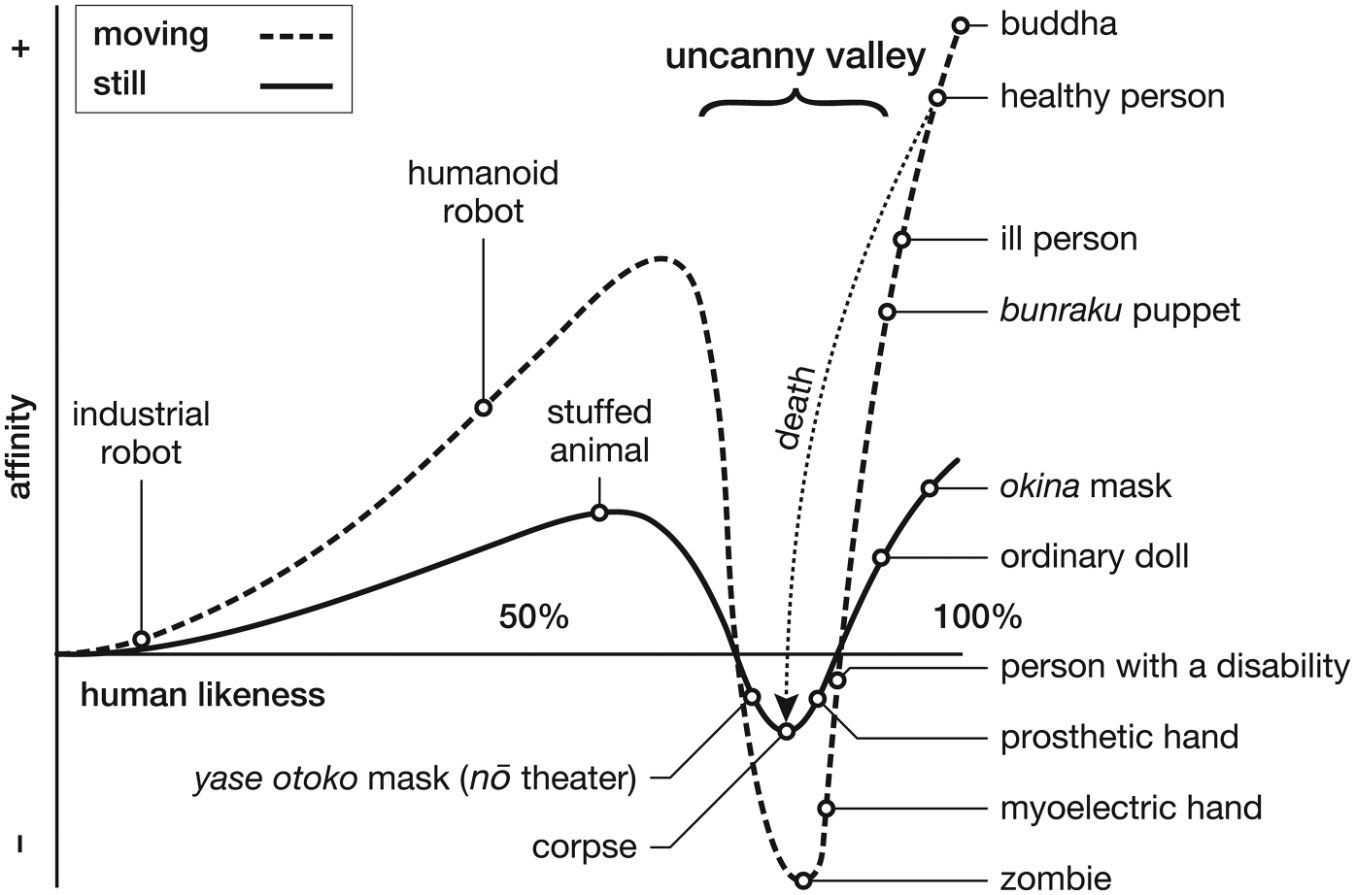
Mori's (2012) uncanny valley plots the relation between an entity's human likeness and the affinity it evokes. Movement steepens the slopes. In 2005, Mori proposed in an unpublished comment adding the face of a Buddhist statue to his graph.

Mori's essay describes how our affinity for robots varies with their human likeness. Industrial robots, designed only to perform specific functions, do not resemble the factory workers they replace, and most people feel little affinity for them (Figure 4). By contrast, toy robots, which have a head, torso, and limbs, delight children. Humanoid robots, which imitate human interaction using sophisticated sensors and actuators, can be even more engaging. Thus, with each human feature added, should we not expect our affinity for robots to increase?

Presumably, if a robot could be built that appeared—in every aspect—identical to a healthy, active person, it would engender even greater affinity. But Mori observes that this goal is perilous. As the robot approaches but fails to become indistinguishable from human, our emotional response abruptly turns from affinity into revulsion. Mori illustrates this point with two thought experiments. In the first, a woman reaches out to shake what looks like a human hand but is, in fact, a myoelectric hand. The discrepancy arouses uncertainty about whether a living being is animate. In the second, a man rummages around lifeless mannequins that suddenly awaken. This aberration sparks uncertainty about whether nonliving objects are inanimate. Jentsch (1997) identifies uncertainty of either kind as eliciting an uncanny sensation.^14^ Mori independently attributes this sensation to the “uncanny valley phenomenon,” the title of his essay.

**Figure 5.**
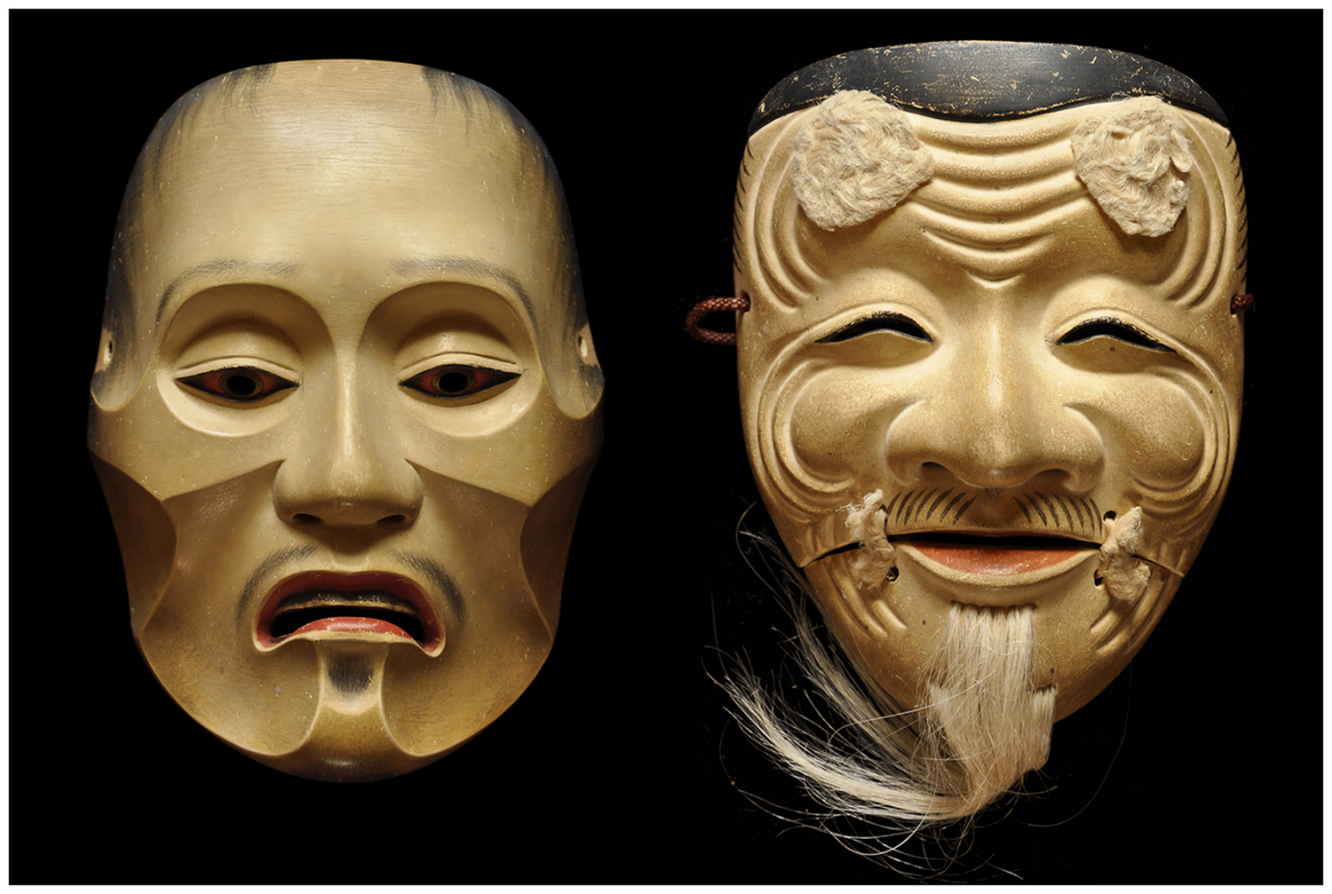
In *nō* theater, the *yase otoko* (emaciated man) mask represents a ghost from hell (left). The *okina* mask represents an old man (right). The masks shown here were carved by Ichiyu Terai in Kyoto, Japan (Photo credit: Shuhei Terai).

To interpret Mori's essay, Chikamatsu's distinction between conceptual and surface realism is invaluable. A blending of these concepts appears in Mori's placement of items along his graph's human likeness dimension. For example, the *okina* (old man) mask from *nō* theater is highly stylized—with a broad nose, closed eyes, and evenly spaced wrinkles, symbolizing wisdom, longevity, and prosperity. In its *surface* realism, it does not appear more human than the *yase otoko* (emaciated man) mask (Figure 5). Nevertheless, Mori's graph presents the *okina* mask as almost human, much more so than the *yase otoko* mask. The placement of the masks only makes sense when one considers their degree of *conceptual* realism: The *yase otoko* mask represents a ghost from hell, which is indeed less human and more eerie than an old man.

**Figure 6.**
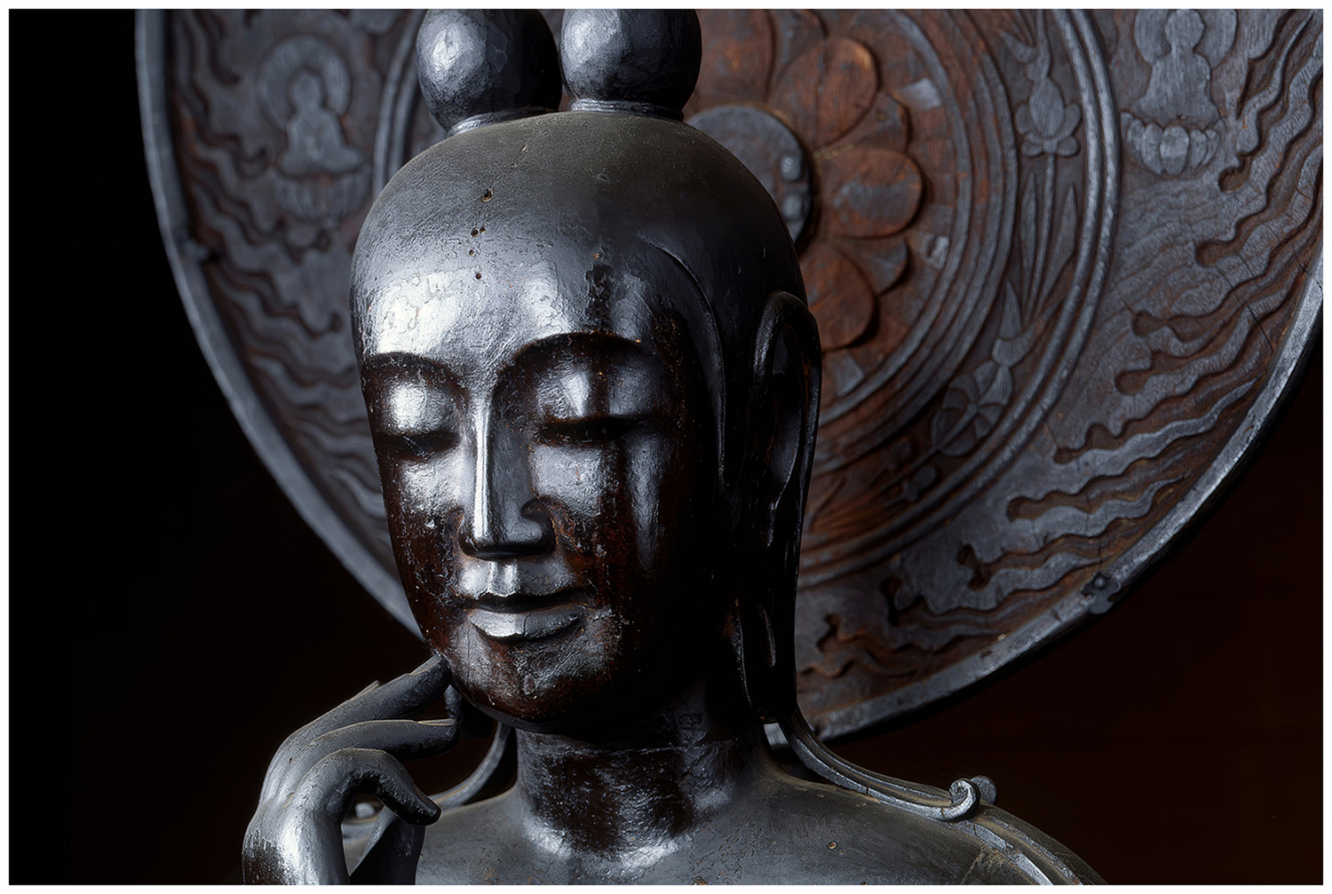
*Miroku Bosatsu* (Bodhisattva Maitreya), a lacquered statue carved from camphor wood, is located at Chūgū-ji, a temple in Kyoto, Japan. The statue dates from the Asuka period (538–710) (Photo credit: Nara National Museum).

In an unpublished 2005 comment, Mori indicates that the face of a Buddhist statue could be positioned above and to the right of the healthy person in his graph (see Appendix; MacDorman, 2019b; Mori et al., 2012). In particular, he mentions three statues of Bodhisattva Maitreya, regarded as our world's next buddha, located at temples in Kyoto and Nara, Japan (e.g., Figure 6). In placing the statue at the pinnacle of human likeness, Mori emphasizes the *concept* the statue embodies—buddha as the *ideal* human—over its physical appearance. This further illustrates how his graph prioritizes conceptual realism over surface realism.

A similar blending of conceptual and surface realism occurs on the curve for moving entities. Mori (2012) observes that on close inspection, *bunraku* puppets appear less real than prosthetic hands. However, when animated on stage, their performance appears so human in their aesthetic mastery as to captivate the audience. Thus, his graph plots a *bunraku* puppet as more human than the prosthetic hand—or even its limbless user. As Chikamatsu surmised, the puppets are engaged in art, which is more beguiling than reality. I depict this interpretation in Figure 3 by making the first peak higher than the second. The audience feels affinity for the puppets, not just because their movements appear human but because each puppet embodies an individual identity (LePage, 2021). The puppet achieves this by portraying its character and the character's relationships in a meaningful story (MacDorman & Cowley, 2006).

## How Narrative Empathy Falls Apart

Narrative empathy refers to processes that enable the audience to identify with and feel sympathy for a character and experience transportation into the character's world. I have proposed that an artificial entity's surface realism disrupts narrative empathy and, thus, the enjoyment of the visual narrative (MacDorman, 2019a). As Chikamatsu observed from the court lady's encounter with her lover's statue, surface realism can turn affinity into eeriness. Although affinity tends to increase with an artificial entity's human likeness and perceived sentience, when a human-looking entity violates human norms, this elicits the uncanny valley effect (MacDorman, 2024).

Zillmann and Cantor's (1977) affective disposition theory predicts that a viewer's emotional responses to a character are shaped by moral judgments about the character's actions. If the actions are deemed benevolent or justifiable, the viewer will come to regard the character favorably, and the viewer's emotional responses will align with the character's emotions (Raney, 2004). Conversely, if the actions are deemed malevolent, the viewer will come to regard the character unfavorably, and the viewer's emotional responses will misalign with the character's emotions.

I proposed empathy disruption theory, which predicts that heroes will be most vulnerable to the uncanny valley's effects (MacDorman, 2019a). This is because, unlike with villains, the audience needs to identify with the hero or at least feel empathy. Otherwise, the visual narrative cannot function as a story. In addition to traditional heroes, we can feel affinity for antiheroes, even a rogue like Shakespeare's Falstaff or a murderer like Chikamatsu's Yohei, both of whom fell victim to circumstances (Gerstle, 1996; Raney, 2004). Because context determines our affinity for a character, it also determines the character's vulnerability to the uncanny valley and, in turn, our feelings about the performance. The impact of context can be generalized beyond the dramatic arts to any relatable character, whether in computer animation, virtual and augmented reality, social and humanoid robotics, or real life.

In sum, affinity depends on many factors apart from human likeness: whether the entity embodies an individual identity, enacts a story, or qualifies as a hero, antihero, or villain, the particulars of the story, whether it makes sense, has artistic merit, and so forth.

## Applying Chikamatsu's Theory to Social Agents

To test empathy disruption theory, we set up an online interactive scenario where participants role-played as a patient interacting with a doctor (MacDorman, 2019a). The theory predicts that an artificial entity's surface realism can disrupt emotional empathy, particularly for heroes, by eliciting the uncanny valley effect, thus reducing narrative enjoyment. The experiment has a 2 × 2 × 2 between-groups design where the doctor is either a hero or villain, the narrative has either a happy or sad ending (the doctor is awarded a fellowship or sued for malpractice), and the doctor and his office are either depicted using computer animation or a real actor. The computer-animated doctor and office were digital doubles of the real ones.

Many effects were as predicted: participants had greater emotional empathy for the hero than the villain. They also had greater sympathy, cognitive perspective-taking, and narrative enjoyment. Participants preferred just endings (hero rewarded, villain punished) to unjust endings (hero punished, villain rewarded). Some effects were counter to prediction. Participants enjoyed the computer-animated scenario more than the real one, even though the computer-animated doctor rated eerier.

A key finding is that when tragedy befell the hero depicted by a real actor, empathy had a strong direct effect on narrative enjoyment (β = .55) but only a small-to-medium-sized effect (β = .25) when tragedy befell the computer-animated hero. This indicates that, for the computer-animated hero, eeriness disrupted the positive effect of empathy on enjoyment, but this effect was washed out by other factors, such as the strong direct effect of transportation on narrative enjoyment in the animated condition. In contrast, this pattern did not occur for the computer-animated hero with a happy ending.

These results align with Chikamatsu's theory of realism, particularly his emphasis on conceptual realism over surface realism. Chikamatsu argued that a work's emotional impact arises from how it conveys meaning and evokes empathy, not from a perfect reproduction of reality. For the animated scenario, transportation may have had a stronger effect on enjoyment because it was more plausible that the animation was a real-time response, unlike the obviously prerecorded video of the human actor. However, the eeriness of the computer-animated character suppressed the effect of empathy on enjoyment in the tragic condition. As Chikamatsu observes, mere surface realism, an overly accurate portrayal of a human without capturing the underlying soul or expressiveness that makes characters relatable, can evoke eeriness. Conceptual realism, which prioritizes emotions and character motivations, is more effective in engaging an audience.

The interactive doctor–patient scenario is not representative of entertainment in general. Eeriness may disrupt narrative empathy differently across genres, such as romance, or in another medium, such as physical robots.

## Lessons for Social Robotics

Chikamatsu's principles for *jōruri* can be adapted to the experience design of social robots:

*Anthropomorphism*'s value to *jōruri* was illustrated in the passage about the envious pine. In social robotics, Paro, modeled after a baby harp seal, leverages its zoomorphism to engage users (Sone, 2017). The robot turns its head toward sources of sound, feigning attention. This trick enables Paro to tap into psychological mechanisms that cause us to attribute human intentions to this mindless robot (Turkle, 2007).

*Individuation* of character types and roles in *jōruri* translates into social robots that exhibit context awareness when interacting with different types of people (e.g., customer vs. employer or adult vs. child). They express their identity, purpose, and function through their overall performance (LePage, 2021; Sone, 2017).

“Show, don’t tell,” according to Chikamatsu, indicates meaning should arise through the story, not its narration. Thus, a social robot should reveal its inner states through its deeds and relationships, not just its statements (MacDorman & Cowley, 2006).

*Simplicity* is essential to creating an interaction flow that feels organic. In *jōruri*, that involves the words following the melody, and in social robotics, it could involve the melody following the emotional prosody of speech (Savery et al., 2021).

*Symbolism* in kabuki is exemplified by colored grease paint, indicating a character's nature, status, and emotions. Similarly, the original Sony Aibo, a robotic pet dog, uses colored LEDs, tail wags, and body movements. Kabuki and *nō*'s symbolism and stylization appear in robot performances in Japan; they encourage audiences to suspend disbelief and perceive the robots as magical, more than machines (Sone, 2017).

*Sympathy* for a character is elicited in *jōruri* and social robotics by aligning the character's actions to the viewer's values (MacDorman, 2019a).

The *uncanny valley* warns contemporary designers not to aim for perfect human likeness but instead for stylized, emotionally engaging designs (Mori, 2012). Robots designed with surface realism—attempting to mimic human appearance exactly—can fall into the uncanny valley due to their lack of perceived authenticity. Instead, robots that focus on conceptual realism, with symbolic or stylized features, can evoke stronger emotional connections without risking the eeriness of accurate but soulless representations.

Though three centuries have passed since Chikamatsu shared his theory of realism with his friend Hozumi, his views on *jōruri* and kabuki can inform how we design robots and other technologies that present themselves as social agents. The goal, to breathe life into a soulless contrivance, remains the same, as does the path forward: to design robots that enact compelling, conceptually realistic stories, whether these robots appear human or not.

## Notes

1. The family name-before-given name convention in Japanese is followed throughout.

2. Macrons indicate long vowel sounds.

3. In Mathur et al. (2020), 358 observers rated 182 robot and human headshots, and in Kim et al. (2022), 539 observers rated 251 robot full-body shots.

4. A functional magnetic resonance study found that the ventromedial prefrontal cortex encoded lower likability for highly humanlike artificial agents, with the amygdala linked to rejection signals, while the temporoparietal junction encoded increasing human likeness (Rosenthal-von der Pütten et al., 2019).

5. *The Tale of Genji*, the world's first novel, describes life at the Heian court (Murasaki, 2008).

6. The blossoms of this citrus tree are fragrant, and the fruit resemble mandarin oranges.

7. This refers to *michiyuki*, lyrical descriptions of scenery during a journey.

8. Matsushima Bay's pine-covered islands and Miyajima Island's shrine and floating *torii* gate are among the three most admired views in Japan.

9. *Waka* and *haikai* are classical and comic Japanese poetry.

10. Despite this claim, Chikamatsu often followed the 5–7 pattern.

11. In classical literature, *aware* denotes any moving experience (Motoori, 2013).

12. Okamoto Bunya, 1633–1694, was an early *jōruri* playwright.

13. Yang Guifei, 719–756, was an imperial consort and one of the four beauties of ancient China.

14. Mori conceived the uncanny valley independently of Jentsch (1997) and Freud (2003), as reported in Jochum and Goldberg (2015).

## Appendix

### Mori Masahiro's Biography

Mori Masahiro (February 12, 1927–January 12, 2025) was Professor of the Tokyo Institute of Technology, where he led robotics research for nearly two decades. He was also President of the Mukta Institute and Honorary President of the Robotics Society of Japan. Mori completed his doctorate in engineering at the University of Tokyo and served on its faculty, among others. Mori is widely celebrated for introducing the concept of the uncanny valley in 1970, which influenced disparate fields ranging from humanoid robotics and computer animation to perceptual psychology and cognitive neuroscience. He is known for his thoughts on robotics and Buddhism, as detailed in *The Buddha in the Robot* and other books. He contributed greatly to robotics research and education, founding some of the earliest robotics contests. He won the NHK Broadcasting Culture Award in 1992, the Medal of Honor in 1994, the Order of the Rising Sun in 1999, and numerous awards from learned societies.

### Mori Masahiro's 2005 Comment

Mori prepared the following comment, dated August 18, 2005, to be delivered at the Views of the Uncanny Valley workshop, held on December 5–7, 2005 at the fifth *IEEE-RAS International Conference on Humanoid Robots* in Tsukuba, Japan:

It is my great honor and pleasure to learn that a workshop on the uncanny valley, a notion I proposed 35 years ago, was going to address the topic in fields as diverse as neuroscience, pattern recognition, artificial intelligence, psychology, and sociology. Unfortunately, I must chair another meeting, which prevents me from being here today. But to make up for my absence, I will make two brief comments on this subject:

1. A dead person's face may indeed be uncanny: it loses color and animation with no blinking. However, in my experience, it can sometimes give us a more comfortable impression than the face of a living person. Dead people are free from the troubles of life, and I think that is why their faces look so calm and peaceful. In our mind, there is always an antinomic conflict that if you take one thing you will lose another. Such a conflict appears on our face as troubles and expressions of discomfort. But when a person dies, he or she is released from this antinomy and has a quiet expression. If so, then where should we position this face on the curve of the uncanny valley? This issue is one of my current interests.
2. I once positioned healthy human beings at the curve's highest point, on the right-hand side of the uncanny valley. Recently, however, I came to think that there is something more attractive and amiable than human beings still farther to the right. It is the face of a Buddhist statue as the artistic expression of the human ideal. You will find such a face, for example, in the Crowned Maitreya of Koryu-ji Temple in Kyoto, the Maitreya of Chugu-ji Temple in Nara, and the Chandraprabha of Yakushi-ji Temple in Nara. Those faces are full of elegance, beyond the worries of life, and have an aura of dignity. I think those are the very things that should be positioned at the highest point of the curve.

Though I introduced the notion of the uncanny valley, I have not examined it closely so far. I hope the two points mentioned above will help advance research on the topic.
